# FOVEA: a new program to standardize the measurement of foveal pit morphology

**DOI:** 10.7717/peerj.1785

**Published:** 2016-04-11

**Authors:** Bret A. Moore, Innfarn Yoo, Luke P. Tyrrell, Bedrich Benes, Esteban Fernandez-Juricic

**Affiliations:** 1 Department of Biological Sciences, Purdue University, West Lafayette, IN, USA; 2 Department of Computer Graphics Technology, Purdue University, West Lafayette, IN, USA

**Keywords:** Fovea, Retina, Foveal pit, Comparative studies

## Abstract

The fovea is one of the most studied retinal specializations in vertebrates, which consists of an invagination of the retinal tissue with high packing of cone photoreceptors, leading to high visual resolution. Between species, foveae differ morphologically in the depth and width of the foveal pit and the steepness of the foveal walls, which could influence visual perception. However, there is no standardized methodology to measure the contour of the foveal pit across species. We present here FOVEA, a program for the quantification of foveal parameters (width, depth, slope of foveal pit) using images from histological cross-sections or optical coherence tomography (OCT). FOVEA is based on a new algorithm to detect the inner retina contour based on the color variation of the image. We evaluated FOVEA by comparing the fovea morphology of two Passerine birds based on histological cross-sections and its performance with data from previously published OCT images. FOVEA detected differences between species and its output was not significantly different from previous estimates using OCT software. FOVEA can be used for comparative studies to better understand the evolution of the fovea morphology in vertebrates as well as for diagnostic purposes in veterinary pathology. FOVEA is freely available for academic use and can be downloaded at: http://estebanfj.bio.purdue.edu/fovea.

## Introduction

The retina is an intraocular neural tissue where photoreceptor cells capture and convert photons of light into an electrical ‘image’ that is sent to the brain. Retinal specializations are regions within the retina where distinct cell populations or structural modifications enhance visual information gathering (e.g., high visual acuity). One of the most studied retinal specializations is the fovea, which is characterized by a high density of cone photoreceptors and a pitted invagination of the retinal tissue that allows for higher cell packing (Slonaker, 1897; Walls, 1937; Pumphrey, 1948). Although the density of retinal ganglion cells increases towards the fovea, at the very center of the foveal pit, the ganglion cell density decreases (and in some cases it is practically zero) as the inner retinal layers are displaced by the tissue invagination (Walls, 1937; Pumphrey, 1948).

The fovea is considered to provide the highest visual resolution of all retinal specializations (e.g., Ross, 2004; Moore et al., 2012). This may be explained by (1) the higher density of photoreceptors at center of the foveal pit (Walls, 1942), (2) the displacement of the retinal layers and vasculature in the foveal pit that facilitates the passing of light (Walls, 1937; Weale, 1966; Martin, 1986; Provis et al., 2013), and (3) various optical effects caused by the steepness of the foveal walls, the curvature of the foveal pit, and the refractive index of the vitreo/retinal boundary (Pumphrey, 1948; Harkness & Bennet-Clark, 1978; Steenstrup & Munk, 1980). These three factors are hypothesized to increase the ability of the fovea to magnify the image (Walls, 1937; Snyder & Miller, 1977; Snyder & Miller, 1978; Williams, 1980), aid in image fixation (Pumphrey, 1948), detect movement (Bloch & Martinoya, 1982; Martinoya, Rivaud & Bloch, 1983), reduce chromatic aberration (Rodieck, 1973), reduce light scattering (Walls, 1937; Martin, 1986; Weale, 1966), and act as a directional focus (Harkness & Bennet-Clark, 1978) and depth (Steenstrup & Munk, 1980) indicator.

Interestingly, the shape and size of the foveal pit vary greatly across species from shallow (concaviclivate) to deep (convexiclivate) (Walls, 1937; Pumphrey, 1948). Deeper foveae are regarded as having higher visual resolution than shallower fovea (Inzunza, Bravo & Smith, 1989; Ross, 2004), as exemplified in raptors (Fox, Lehmkuhle & Westendorf, 1976; Reymond, 1987; Gaffney & Hodos, 2003). However, the quantitative characterization of the contour of the foveal pit has received relatively little attention from a comparative perspective. Williams (1980) attempted to measure the contour of the foveal pit using psychophysical techniques. Ophthalmoscopic reflex techniques have been used to measure the human foveal pit curvature (O’Leary, 1985; Gorrand & Delori, 1999; Van & Roorda, 2003). More recently, retinal, and in many cases foveal, architecture *in vivo* has been studied using optical coherence tomography (OCT; e.g., Hammer et al., 2008; Dubis, McAllister & Carroll, 2009; Ruggeri et al., 2010; Knighton & Gregori, 2012). Dubis, McAllister & Carroll (2009) and Scheibe et al. (2013) developed algorithms that enabled the extraction of several different morphological dimensions (slope, depth, and diameter) from OCT scans. The application of these techniques to vertebrates of different sizes and morphologies could be quite challenging (i.e., small vertebrates may be difficult to handle). Additionally, although OCT technology has become available in many ophthalmology departments, its availability may still be rather limited in basic biology units. However, there are some archives of histological cross sections of the retina from multiple species of vertebrates that may be used as sources of foveal morphology data. An example is the Comparative Ocular Pathology Lab of Wisconsin, which houses >23,000 paraffin-embedded and sectioned eyes from hundreds of different species (http://www.vetmed.wisc.edu/pbs/dubielzig/pages/coplow/main.html).

One of the issues with the comparative study of fovea morphology is the lack of a standardized methodology to measure the contour of the foveal pit in different vertebrates (i.e., varying in eye size). We developed a new piece of software that automatically measures different foveal parameters (width, depth, and slope of the foveal pit) using images from histological cross-sections as well as those from OCT scans. We explain how the program works, present new data on the foveal morphology of two Passerine bird species based on histological cross-sections, and assess its performance relative to previously published OCT-generated images. By characterizing the morphology of the fovea across species with a standardized method, we will be in a stronger position to better understand the function of foveal vision in different species, the evolution of this retinal specialization in relation to different ecological traits, and the detection, prognosis and epidemiology of some ocular diseases in vertebrates.

## Methods

We captured 3 house sparrows (*Passer domesticus*) and 3 white-crowned sparrows (*Zonotrichia leucophrys*) in Tippecanoe County, Indiana using hanging finch traps, house sparrow traps, and mist-nets. We housed birds in 0.61 × 0.61 × 0.76 m cages in the animal facilities at the Purdue University campus under a 12 h light/dark cycle. All animal procedures were approved by the Purdue Animal Care and Use Committee protocol 09-018.

### Tissue processing

After euthanasia, we removed and hemisected the eye anterior to the ora serrata with a razorblade, and removed the vitreous humor with tweezers and scissors. The hemisected eye cup was immersed in Bouin’s fixative for 24 h, and later washed in 0.01 M PBS. We identified the retinal area with the fovea, cut a 2 mm thick strip from the eyecup, and placed it in 70% ethanol for seven days to remove the Bouin’s fixative. We embedded the section of the retina containing the fovea in paraffin wax, and serial sectioned along the anterior–posterior axis with a Thermo Scientific Shandon Finesse ME microtome (Thermo Scientific, Waltham, MA, USA). Serial sectioning was performed at 20 µm intervals until the fovea was identifiable by examination with a 10× ocular, upon which serial sectioning was performed at 5 µm intervals until the opposite side of the fovea was reached. We stained the tissue with haemotoxylin/eosin in a Thermo Scientific Shandon Varistain 24-3. Stained sections were evaluated using an Olympus BX51 microscope. The section containing the center of the fovea was identified as the section with the deepest foveal depth and displacement of retinal ganglion cells from the center of all sections considered. A photograph was then taken of the center section with an Olympus S97809 microscope camera. Because the foveae are usually not completely circular, we consistently used the same axis and direction (nasal-temporal) to make the cuts from each single retina to minimize bias.

### Fovea image processing

Photographs of the stained cross-sections were captured with SnagIt (www.techsmith.com/Snagit) and saved as PNG files for importing into FOVEA software. From papers that measured the depth and width of several human foveae (Hammer et al., 2008; Dubis, McAllister & Carroll, 2009), we used one published OCT scan image from each study to compare the performance of the FOVEA software with that of the OCT software. Specifically, we extracted Fig. 6D from Hammer et al. (2008) and Fig. 2 from Dubis, McAllister & Carroll (2009) using Adobe Acrobat.

Dubis, McAllister & Carroll (2009) calculated foveal depth and width from human samples; but because of the low curvature of the human fovea, they assumed the lower curve to be a flat line. Our approach calculates the retinal depth and width by taking into consideration different degrees of curvatures which makes FOVEA applicable to a wide variety of vertebrate species with different fovea morphology. Consequently, our approach can be considered a generalization of Dubis, McAllister & Carroll (2009).

Appendix S1 provides a step-by-step description of how to use the FOVEA software. FOVEA can process retinal images with different focal distances and resolutions. The distance in pixels can correspond to a different real distance of the corresponding features in the image. The user is asked to convert the pixel distance into a metric distance by selecting a measure at the bottom of the application and typing the actual distance in µm (Appendix S1). The results are provided in both pixels and µm. Additionally, the input images of the retina can be at any angle or color variation.

**Figure 1:**
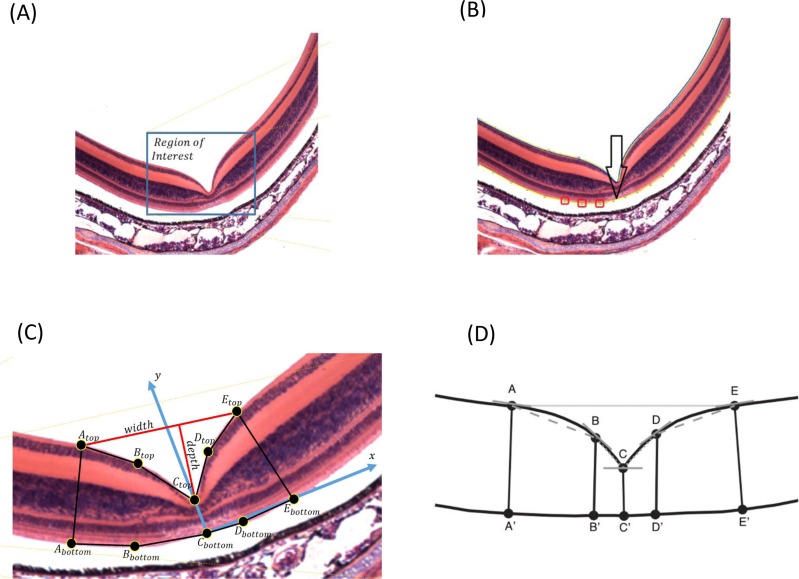
(A) The input to FOVEA is an image of a cross section of retinal tissue. (B) To estimate the foveal pit parameters, there are three key elements: (I) a retina contour (blue) that is automatically detected by the program, (II) control points (red) that are defined by the user, and (III) a reference curve (yellow) that is established by the program. FOVEA establishes different points considering the retina contour and the reference curve (*A*_top_, *A*_bottom_, *B*_top_, *B*_bottom_, *C*_top_, *C*_bottom_, *D*_top_, *D*_bottom_, *E*_top_, and *E*_bottom_) to estimate fovea width and depth (C), and measures the slopes between points A–B, B–C, C–D, D–E (D) as proxies of the inclination of the foveal walls.

Because the input images may have different sizes, the user needs to select the region of interest where the foveal pit is located (Fig. 1A). The image may have additional noise that can be removed using Gaussian filtering, by which a new value of a pixel is calculated as a weighted average of the neighboring pixels by averaging with a Gaussian kernel: $G(x)= \frac{1}{\sqrt{1\pi {\sigma }^{2}}} {e}^{- \frac{{x}^{2}}{2{\sigma }^{2}} }$, where *σ* is the SD of the Gaussian distribution, and *x* is the location of the pixel. We used a 3 × 3 pixel filter size. Additionally, the user can manually select areas that can be erased if some noise is still present (Appendix S1).

FOVEA detects the inner *retina contour* by estimating the color variation of the image from its histogram so that images with different color variation can be processed in a standardized manner (Fig. 1A). The *retina contour* is automatically detected. Optionally, some irregularities caused by tissue processing can be manually smoothed (Appendix S1).

To measure foveal depth, the width of the retina at different locations needs to be established. The user is asked to add a *reference curve* at the “bottom” of the retina by manually adding points along the desired region. Any portion of the retina to be delineated as the reference curve can be selected; however, we suggest to select the outer segment and inner segment interface due to its consistent shape (compared to more inward retinal layers) and lack of distortion from retinal pigment epithelium attachments (compared with the outer segment layer of the photoreceptors). Figure 1B shows the retina contour in blue, the user-defined points as red boxes, and the *reference curve* in yellow. Both the *retina contour* and the *reference curve* are piecewise polynomial curves, whereby end points are defined as the centers of the corresponding pixels.

**Figure 2:**
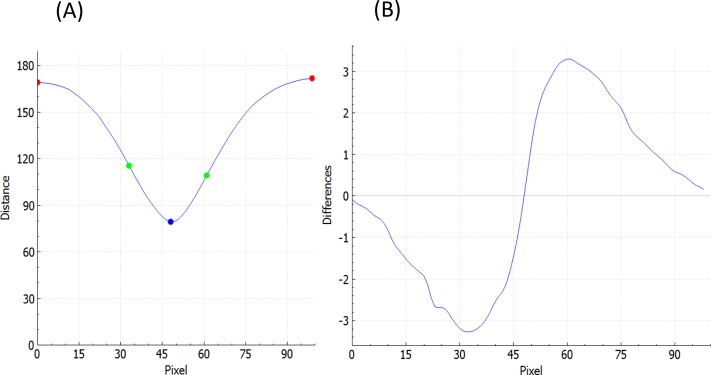
Distance of each point defined in Fig. 1 (*A* and *E* in red, *B* and *D* in green, *C* in blue) on the *retina contour* from the *reference curve* (A) and the derivation of that distance (B).

To estimate fovea width and depth after the *retina contour* and the *reference curve* are established, FOVEA estimates several additional points (*A*_top_, *A*_bottom_, *B*_top_, *B*_bottom_, *C*_top_, *C*_bottom_, *D*_top_, *D*_bottom_, *E*_top_, *E*_bottom_; Fig. 1C, Appendix S1). The depth of the fovea is calculated as the distance of the closest point on the *retina contour* and the *reference curve* (Fig. 1C). By processing both the retinal contour and the reference curve, we extract a *distance graph* (Fig. 2A) and its first derivation called the *differentiate graph* (Fig. 2B). Having the *retina contour denoted by R* as a piecewise linear function connecting pixels and the distance graph denoted by *S*, the differentiate graph *S*′ is calculated by a discrete differentiate operator that approximates the first derivation *dS*∕*dt* by discrete differences $ \frac{\Delta D}{\Delta t} = \frac{S({x}_{i})-S(x)}{\mathrm{dist}{|}{x}_{i},x{|}} $, where *S*(*x*_*i*_) is the distance of pixel *x*_*i*_ from the reference curve, and dist|*x*_*i*_, *x*| is the Eucledian distance of the pixels *x*_*i*_ and *x*.

From the *distance graph* and the *differentiate graph* (Fig. 2), the points *A*_top_, *C*_top_, and *E*_top_ are calculated and their counterparts on the bottom denoted as *A*_bottom_, *C*_bottom_, and *E*_bottom_ are detected. The points *A*_top_ and *E*_top_ are at the maximum distance from the baseline (*x*-axis in Fig. 2A). It is important to note that how these points are established is *independent* of the selection of the window in Fig. 1A. In particular the point *A*_top_ is found automatically by searching the local maxima from the beginning of the baseline towards increasing *x*-values, and *E*_top_ is found automatically by searching the local maximum in the opposite direction starting at the end of the baseline. The points *A*_bottom_ and *E*_bottom_ are also found automatically from the distance graph as the counterparts of *A*_top_ and *E*_top_. The retina is then placed in the center of the coordinate system and de-rotated. More specifically, we place *C*_bottom_ at the center of the coordinate system and the *y*-axis is aligned to the line given by points *C*_bottom_ and *C*_top_. The *x*-axis points to the right (Fig. 1C). Then, the points *B*_top_ and *B*_bottom_, as well as *D*_top_ and *D*_bottom_, are calculated. The top points are found as the inflexion points on the *distance graph* that are the extremes in the *differentiate graph*. The points located at the bottom are found as their counterparts on the lower part from the *distance graph*. After all these points are detected, we can calculate the foveal width, which is the distance between *A*_top_ and *E*_top_. The foveal depth is the perpendicular distance from *C*_top_ to the line given by *A*_top_ and *E*_top_.

Finally, FOVEA calculates the slopes of different points. In particular, it estimates (a) the local slope of all top points as the angle between the tangent vector in a point and the *x*-axis, and (b) the line slopes for the following pairs of points *A*_top_*B*_top_, *B*_top_*C*_top_, *C*_top_*D*_top_ and *D*_top_*E*_top_ (Fig. 1D). On each side of the foveal wall, FOVEA estimates two slopes (in the upper and lower part of the wall) to better characterize the diversity of the inclination of the foveal pit across different species.

### Statistical analysis

Because of the low sample size per species (three retinas belonging to three different white-crowned sparrows, and three retinas belonging to two different house sparrows) and the data not meeting the assumptions of parametric tests even with transformations, we conducted non-parametric Mann–Whitney tests to compare foveal traits between white-crowned sparrows and house sparrows. We also used a single mean versus population mean two-sided tests (StatSoft, 2013) to compare our single estimates of foveal depth and width with the means measured using the OCT software (Hammer et al., 2008; Dubis, McAllister & Carroll, 2009). Our intention was to examine whether the performance of the FOVEA program was comparable to those used in OCT systems.

## Results

**Figure 3:**
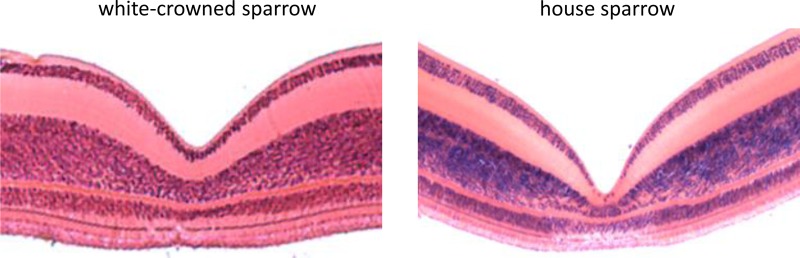
Histological cross sections of the foveae of the white-crowned sparrow (*Zonotrichia leucophrys*) and the house sparrow (*Passer domesticus*).

Using FOVEA, we measured the width, depth, and slopes of the foveal pit of 3 white-crowned sparrow and 3 house sparrow foveae based on histological cross-sections (means ± SE shown in Table 1). We found that house sparrows had deeper foveae and steeper foveal walls than white-crowned sparrows (Table 1 and Fig. 3).

**Table 1:** Means and SE of different foveal parameters of white-crowned sparrows and house sparrows. Between-species differences were tested with the Mann–Whitney test. Significant results are marked with bold.

	White-crowned sparrow	House sparrow	Statistical test
Foveal width (um)	469.91 ± 62.12	607.34 ± 70.58	*Z* = − 1.53; *P* = 0.127
Foveal depth (um)	123.26 ± 11.77	192.54 ± 24.31	*Z* = − 1.96; ***P*** = **0.049**
Slope A–B	0.27 ± 0.04	0.49 ± 0.28	*Z* = 0.22; *P* = 0.827
Slope B–C	0.84 ± 0.35	0.91 ± 0.10	*Z* = − 0.65; *P* = 0.513
Slope D–C	0.74 ± 0.04	0.98 ± 0.07	*Z* = − 1.96; ***P*** = **0.049**
Slope E–D	0.28 ± 0.05	0.42 ± 0.15	*Z* = − 0.65; *P* = 0.513
Slope A–C	0.55 ± 0.17	0.70 ± 0.17	*Z* = − 1.09; *P* = 0.275
Slope E–C	0.51 ± 0.02	0.70 ± 0.07	*Z* = − 1.96; ***P*** = **0.049**

We also measured with FOVEA the width and depth of the human foveal pit from examples of OCT images published in two different papers (Hammer et al., 2008; Dubis, McAllister & Carroll, 2009). The FOVEA estimate of foveal width (1.964 mm) did not differ significantly from the mean (1.97 ± 0.03 mm; *P* = 0.784) obtained in Dubis, McAllister & Carroll (2009). Additionally, the FOVEA estimates of foveal depths, 118.31 µm and 117.61 µm, did not differ significantly from the means reported in Dubis, McAllister & Carroll (2009) (122.53 ± 3.2 µm, *P* = 0.195) and Hammer et al. (2008) (121 ±4.30 µm, *P* = 0.453), respectively. This finding suggests that OCT images can also be used as a source of information for FOVEA.

## Discussion

In the last few years, there has been a surge in the standardization of quantitative tools to study, from a comparative perspective, the vertebrate eye. For instance, Moore et al. (2012) developed a method to quantify the position of the retinal specialization and variation in cell density across the retina using retinal topographic maps, many of which are now available in an online database (http://www.retinalmaps.com.au/; Collin, 2008). Garza-Gisholt et al. (2014) developed a program to generate retinal topographic maps based on cell density counts using different algorithms, and Sterratt et al. (2013) proposed a method to reconstruct the retina based on flattened topographic maps. Our FOVEA program is available for free and provides a user-friendly interface that enables standardized measurements of the inner fovea contour from images obtained from both histological cross-sections and optical coherence tomography (OCT). This can be convenient as OCT has many advantages in terms of access to foveal images *in-vivo*, 3-D representation of the foveal pit, etc. However, classic cross-sections can be obtained for small species, wild species with diseases (e.g., bird flu, West Nile virus, etc.), and aquatic vertebrates, whose fovea morphology may be more challenging to measure with OCT.

FOVEA has some potential drawbacks that are worth pointing out. First, in some retinae, the tissue outside of the fovea is very similar in thickness to the region just prior to the fovea pit (perifoveal region), which can lead to problems when finding the local maximum thickness values. This may be due to a gradual rate of change in cell density from the retinal periphery to the perifoveal region, or it may be due to the change in relative abundance of extrasomal tissue within the retina. However, the depth of the fovea should not be affected by this limitation, because the thickness is very similar throughout the foveal pit itself. Second, the image of the fovea is supposed to have a scale to transform the measurements from pixels to the metric system. Generally, this is not a problem, but there may be some cases of old images without the necessary scale. Third, FOVEA does not account for histological shrinkage; however, fixation during cross-sections is fast so differential shrinkage across the fovea should remain minimal. Finally, FOVEA does not take into consideration the 3-D structure of the fovea as it measures fovea morphology based on a 2-D image selected as the one with the deepest foveal pit after multiple cross-sectioning the fovea. Simplifying 3-D structures into 2-D ones is not unique in comparative visual anatomy, as flattened retinal wholemounting has been used for many decades to study between-species variation in the type and position of the retinal specialization in the vertebrate retina (reviewed in Ullmann et al., 2012). One possibility to address this limitation is by measuring with FOVEA multiple cross-sections from the same fovea and then averaging the output parameters to increase the precision of the estimates. Despite these limitations, we believe that FOVEA can provide accurate data particularly with good quality histological preparations or OCT images.

The resolution of the input images ought to be high enough so that the distance and the differentiate graphs do not suffer from the irregularities caused by noise procedure. Also, the discrete operators calculating the graphs consider a liner window of 3 pixels that are averaged and locally smooth the noise. Therefore, with high resolution input images, the noise can be minimized because of FOVEA noise filtering. As mentioned above, the noise is automatically removed by filtering the images by Gaussian filter of size 3×3 pixels. Moreover, additional local noise filtering is performed by using the discrete operator that calculates the graphs. In some cases, if there is a left over noise in the image, the user can remove it manually (Appendix S1).

From a comparative point of view, the contour of the fovea inner surface has received relatively little attention besides the association between deeper foveae with higher visual acuity (Walls, 1942). For instance, Fite & Rosenfield-Wessels (1975) found between-species differences in the width and depth of the foveal depth of nine species of birds belonging to different Orders, and Reymond (1985) and Reymond (1987) characterized the foveal pit of two species of raptors. Here, we reported quantitative differences in the foveal depth of two species of song birds. This evidence suggests that there may be a large degree of variation in foveal pit morphology that is largely unexplained, with the exception of the work of Fite & Rosenfield-Wessels (1975) showing that species with wider foveal pits tended to have smaller eyes. Even more important is the possibility of assessing in the future how foveal pit morphology may be related to other traits, such as behavior, ecological niche, degree of phylogenetic relatedness, etc. The quantification of the fovea morphology with the standardized algorithm presented here would make these comparative studies possible to better understand the evolution of the fovea in vertebrates.

There are potentially multiple sources of information on the morphology of the retina besides already published accounts. Veterinary pathologists routinely make cross sections of the retina for diagnostic purposes, and both paraffin-embedded and sectioned specimens are often saved for future reference (e.g., Comparative Ocular Pathology Lab of Wisconsin). Additionally, OCT can now be used to detect different retinal pathologies (Tanna et al., 2009; Rauscher et al., 2013), increasing the availability of retinal images that can quantified with FOVEA. We encourage the development of an open online database with fovea images from different taxa along the lines of the retinal topographic map databaset already in place (Collin, 2008). Having this dataset would enable comparisons not only between species, but also within species to better characterize retinal disorders. More specifically, distinguishing between fovea parameters would allow us to predict how morphological differences will result in different optical effects on a light image before it strikes the photoreceptor layer. In turn, this could enhance our understanding of the function of the fovea in different taxa.

## Supplemental Information

10.7717/peerj.1785/supp-1 Data S1 Raw data

10.7717/peerj.1785/supp-2 Appendix S1 Manual for FOVEA software

10.7717/peerj.1785/supp-3 Supplemental Information 1 FOVEA software Files to install FOVEA software.

## Additional Information and Declarations

### Data Availability

The following information was supplied regarding data availability: Raw data has been uploaded as Supplemental Information. FOVEA can be downloaded at http://estebanfj.bio.purdue.edu/fovea.
