# Supplementary material for: FOVEA: a new program to standardize the measurement of foveal pit morphology
Source: PeerJ. 2016 Apr 11;4:e1785. doi: 10.7717/peerj.1785 (PMC4830241; doi:10.7717/peerj.1785)

Appendix 1: Manual for Fovea Software

Fovea is a software to calculate foveal width and depth by detecting local maxima and minima from fovea images in order to estimate foveal depth and width. This document provides instructions for using the software.

Please, unzip and execute fovea.exe. If you get an error message, you can resolve it by installing "Visual C++ Redistributable Packages for Visual Studio 2013" from this website: https://www.microsoft.com/en-us/download/details.aspx?id=40784

When you execute "**fovea.exe**" file, the initial software layout looks like this:

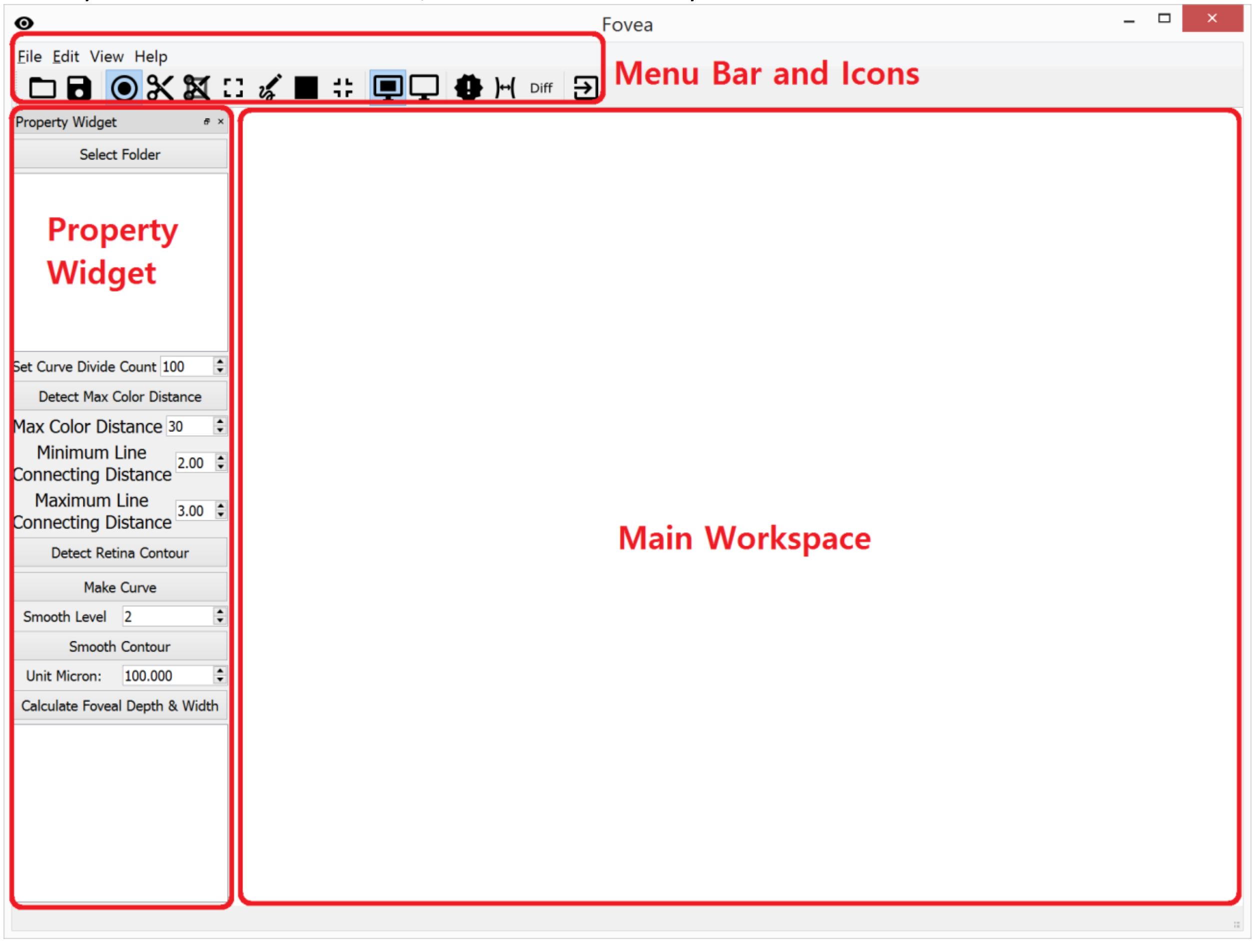

On the top, there is a **menu bar, and icons** are located below the menu. On the left side, there is a **property widget**, which contains buttons and settings for running the detection algorithms. The white blank area is the **main workspace**.

1. Initial Fovea Image Directory

The first step is selecting the directory of fovea images. All images in the directory will be shown. By clicking on one of the red boxes you indicate the fovea image directory:

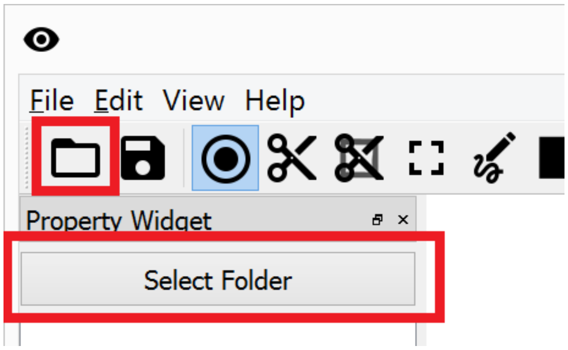

After that, you will see the image files (red box):

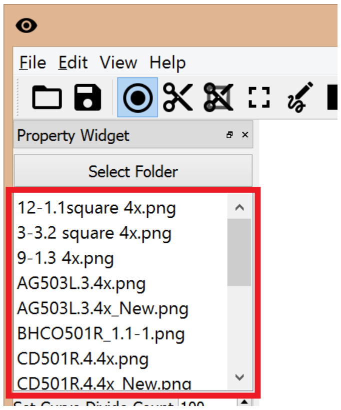

2. Load and View a Fovea Image

After selecting one of the image files, the image is loaded and displayed on main workspace:

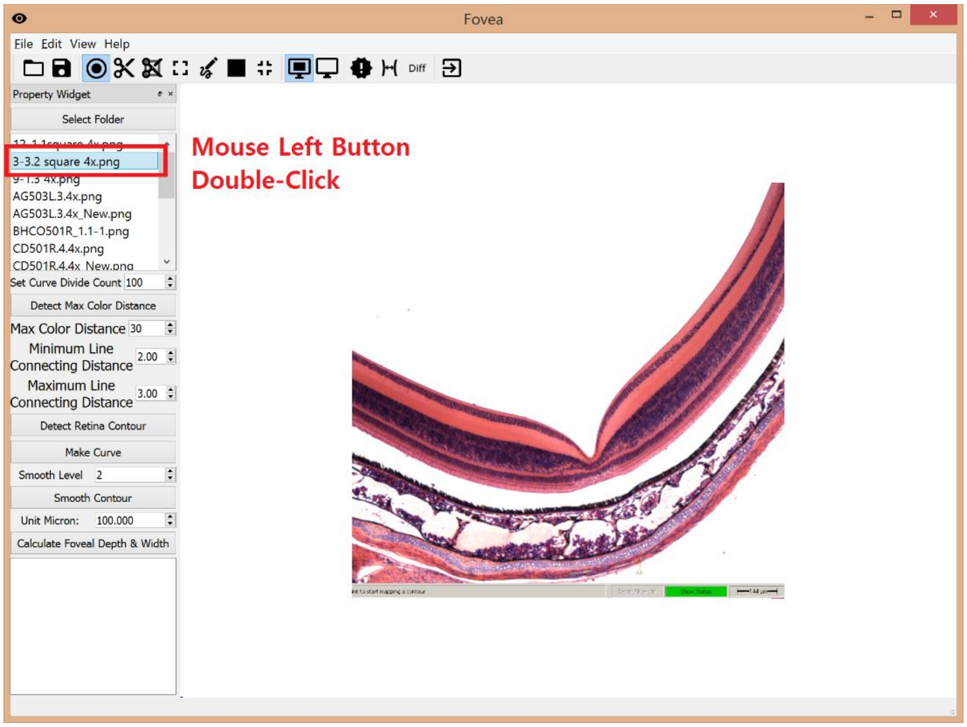

You can maximize the image view (see the red box below the image).

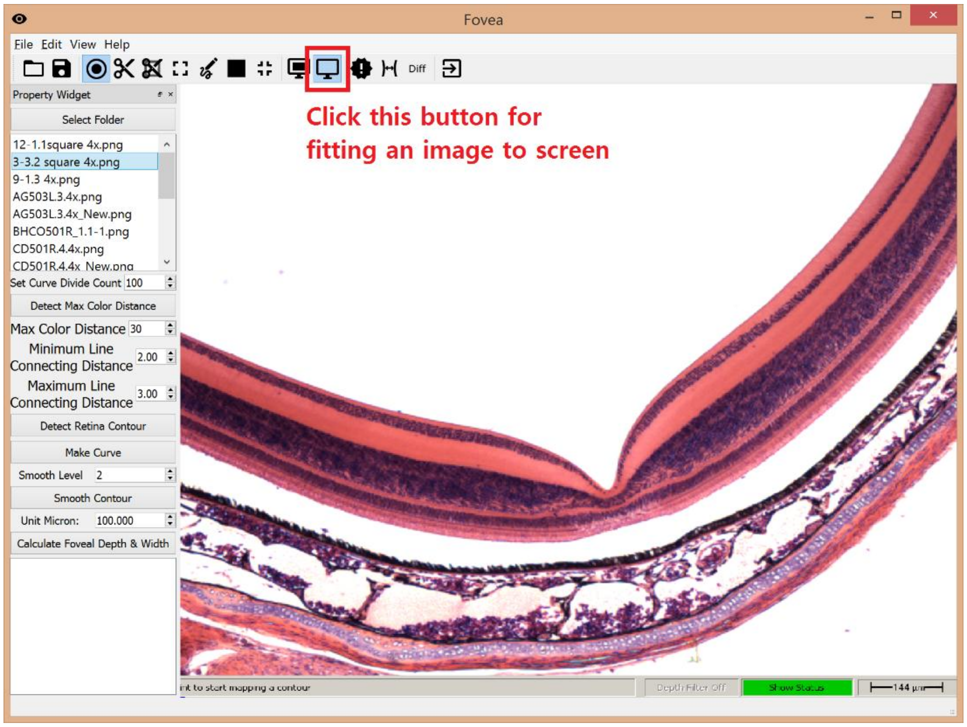

3. Edit image

If the background of the image is too noisy, you may clean it before further processing. There are three options.

First, when you are only interested in a part of image, then you can cut the image.

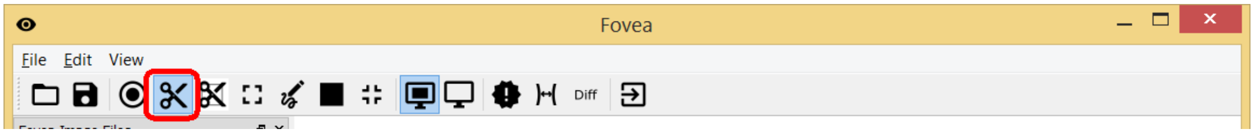

Click the red box icon. And in the workspace, you can **"mouse left click and drag"** the *region of interest* (ROI). Then **click the "Enter" key**.

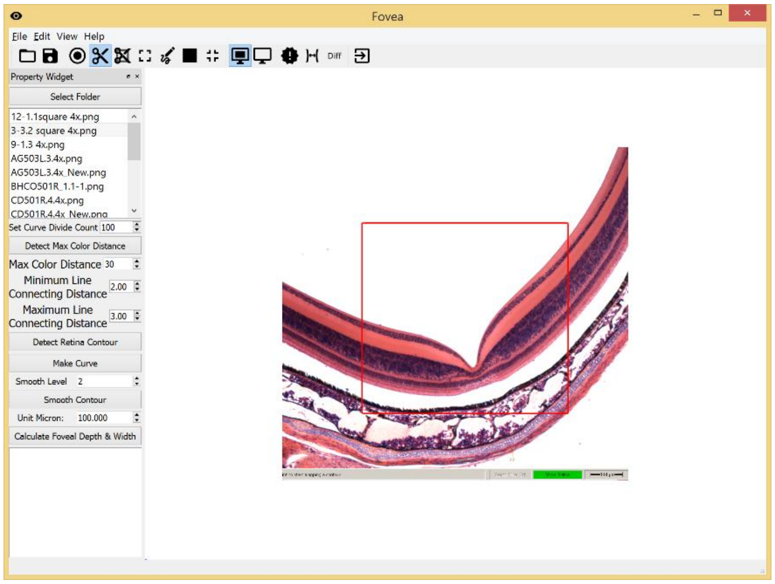

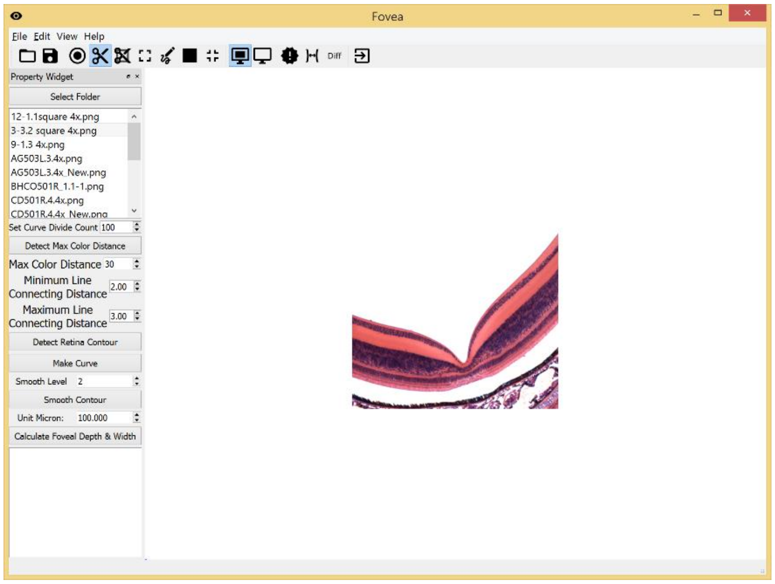

The region of interest stays and the outside of ROI is removed.

If you want to make squared ROI image choose the icon below.

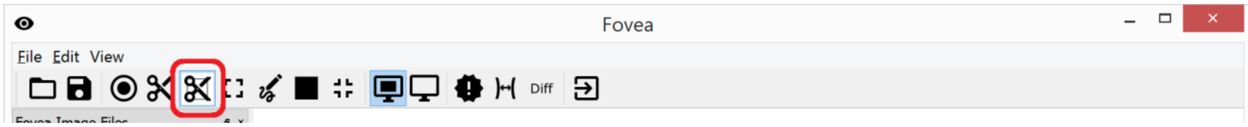

Then, **"mouse-left click and drag"** only creates **square-size ROI** region. Then **click the "Enter" key**.

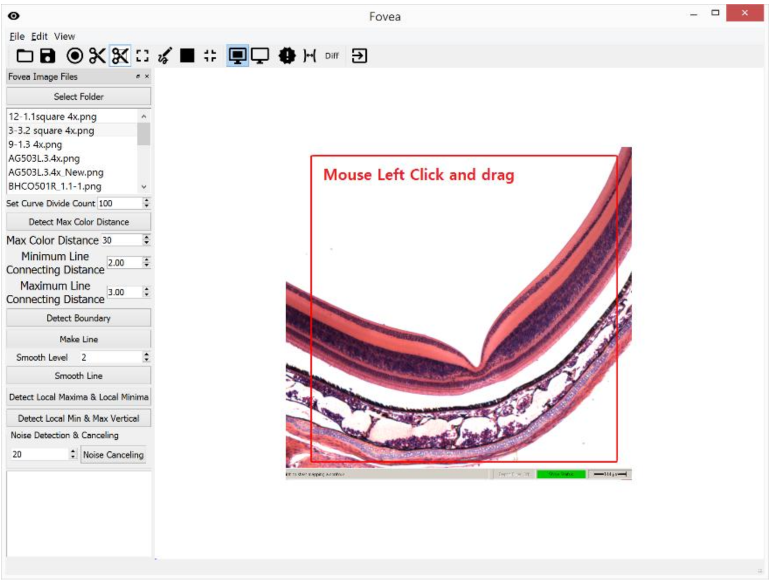

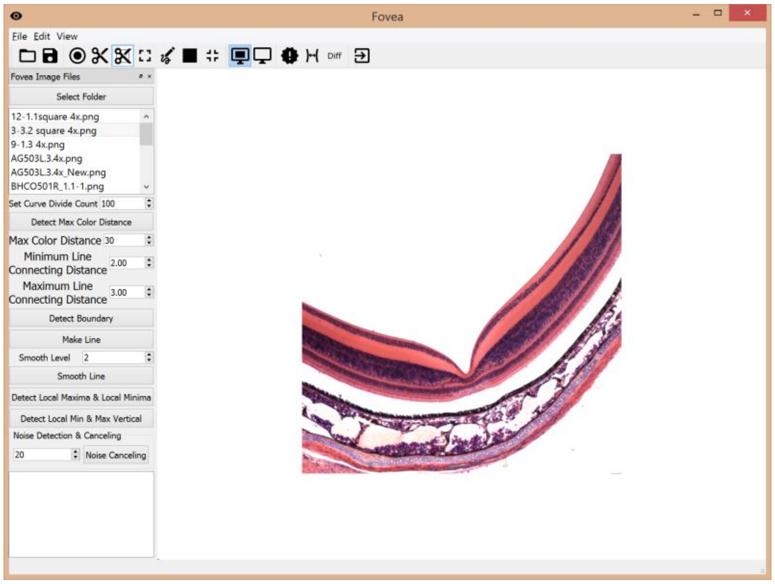

Unnecessary parts of the image can be erased.

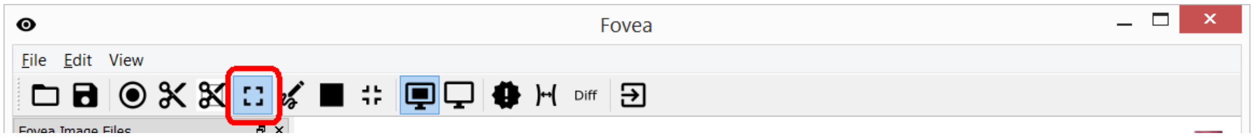

The above icon shows the function to “clear image” icon. Similarly, when you “mouse left click and drag” the region that is not related to fovea, then click “Delete” key in your keyboard. The selected region will be removed. The actual fovea region should not be erased by this functionality.

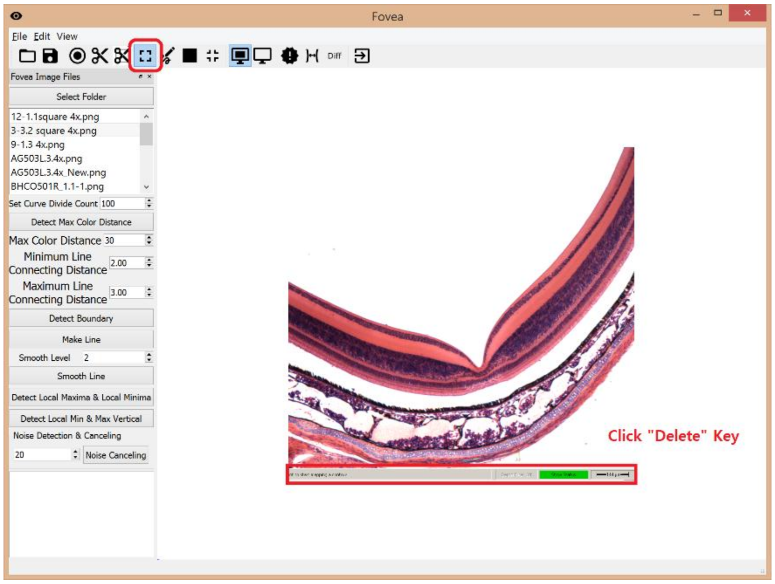

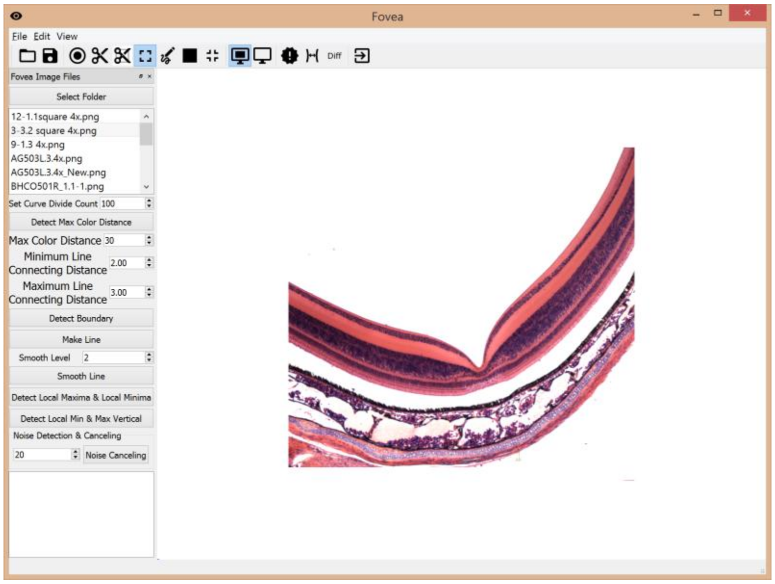

4. Detection of the Retina Contour

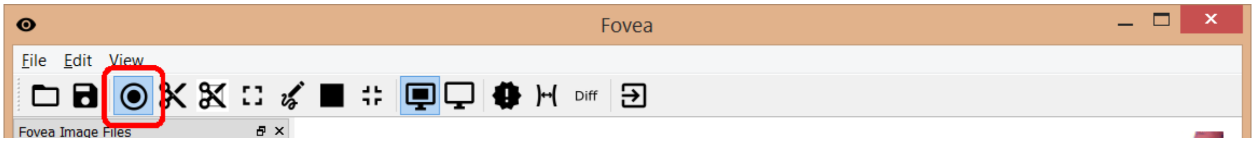

Click above icon, and selected a region (may include a bit of noise). To detect the retina contour correctly, the background color variance (noise) of an image should be calculated first. If the color variance is less than the given value, it will not be considered as boundary edge.

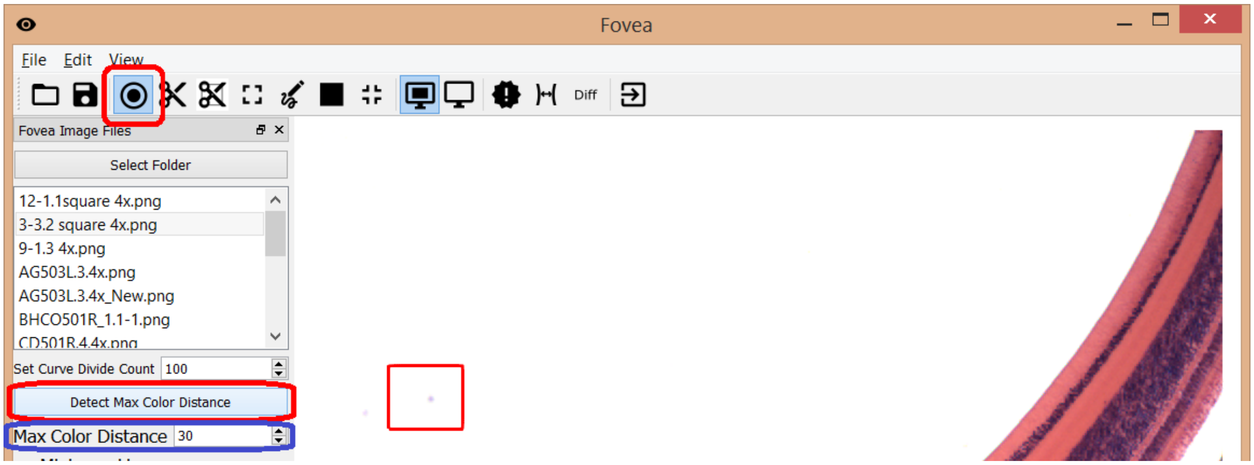

Then click "**Detect Max Color Distance**" button in the Property Widget. Then **"Max Color Distance" (blue box in the image) value, below the button, is changed**. The default value is 30. Suggested value is less than 80, because higher negatively values influence the amount of noise.

Then click "**Detect Retina Contour**" button in Property Widget.

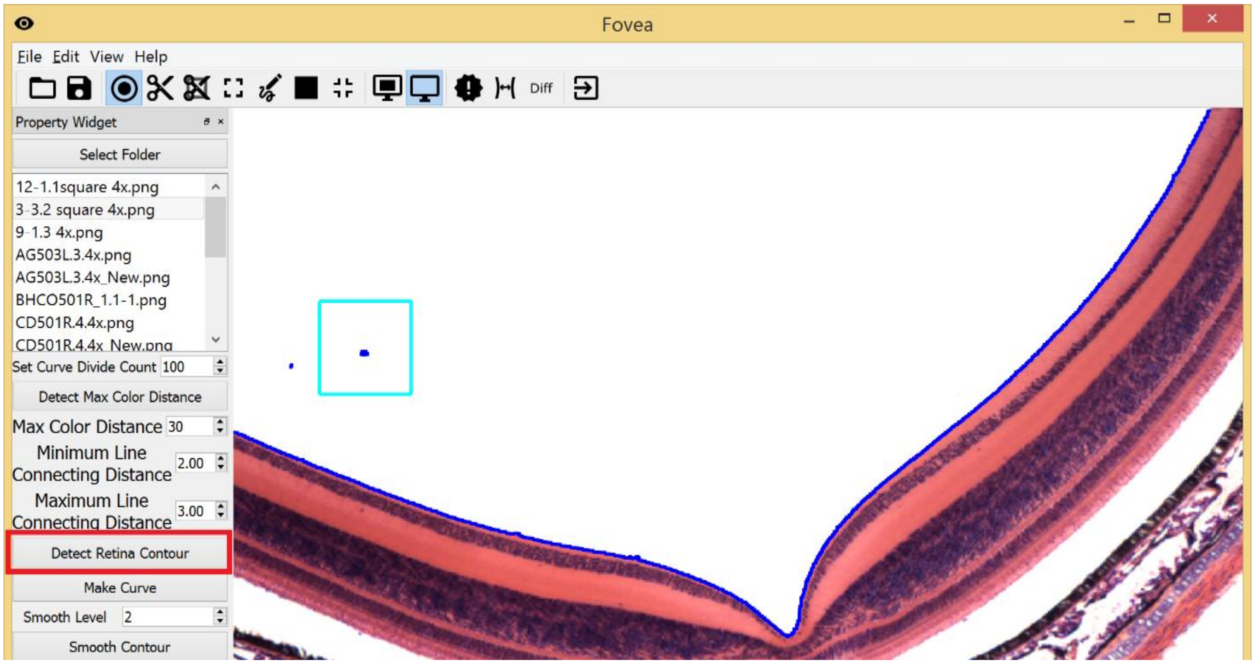

The outline of the retina is detected. However, in this example, two points of noise are also detected. They can be manually deleted by **"Mouse left click and drag the two points"**, and clicking the **"Delete"** key.

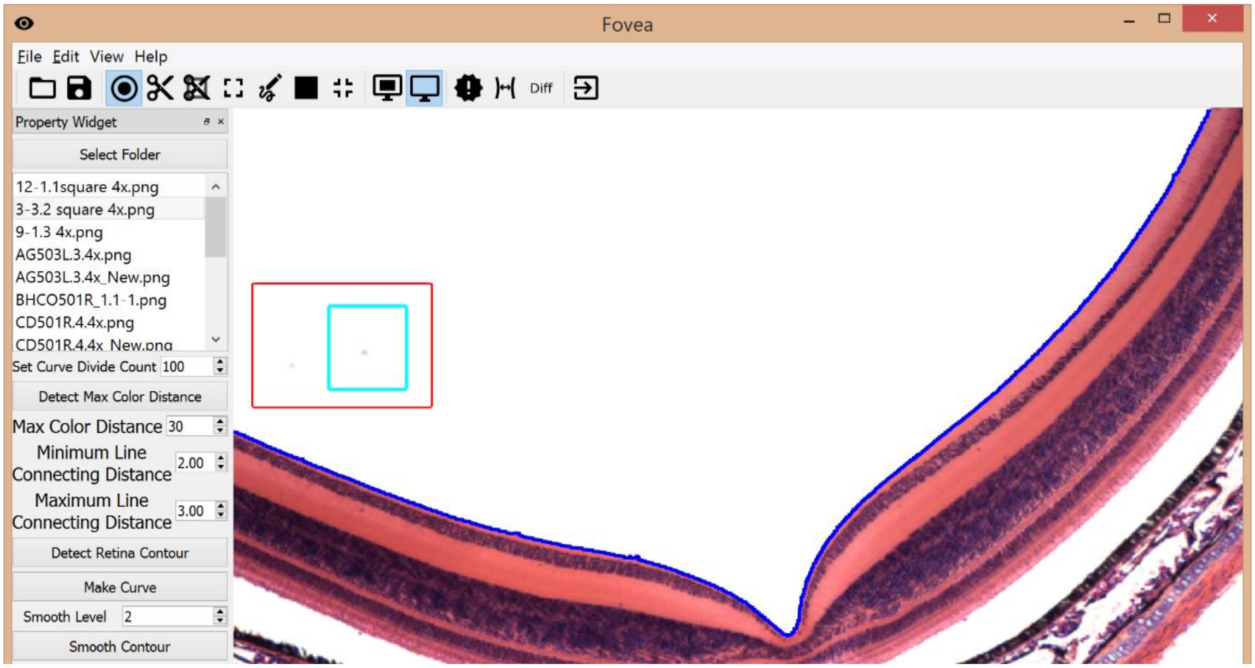

So, the two noise points are erased.

Next, click “**Make Curve**” button in the Property Widget.

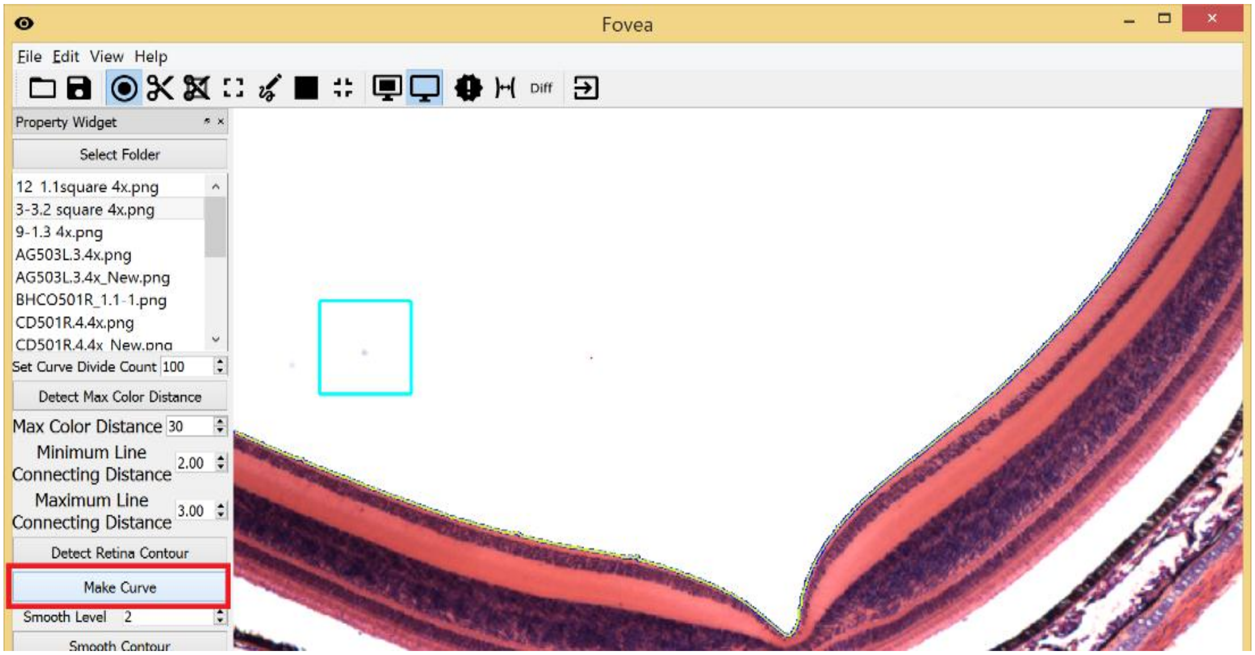

The curve is created, but it may be too rough (as shown in above image, sharp changes in the boundary curve exist). To calculate local minimum and maximum precisely, the curve should be smooth that is achieved by using the function “**Smooth Contour**”.

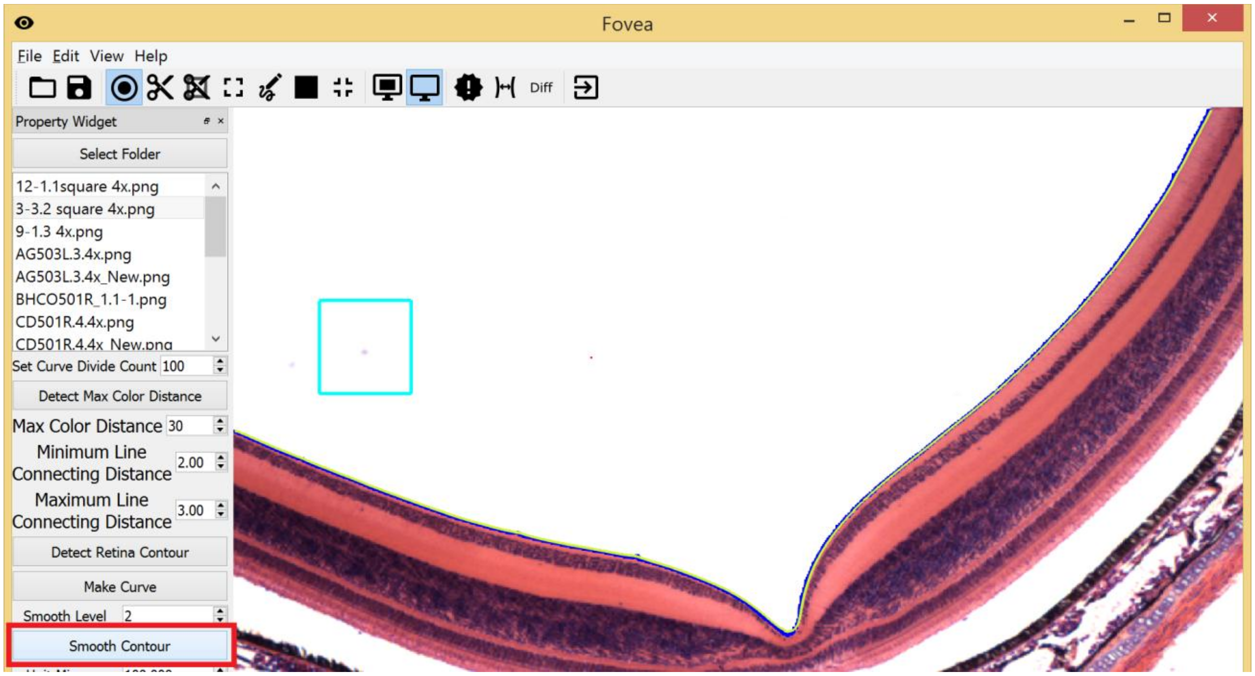

“Smooth Contour” can be applied several times until the boundary line is smooth enough and still fits the real boundary of the retina. In the image above, the smoothing has been applied 3x. In general case, applying 2-3x is enough.

5. Adding a Reference Curve

In order to detect local maxima and minima from the fovea images, a piece-wise linear reference to the boundary line is needed. The foveal depth and width are based on **the distance from the reference curve to the detected boundary line**. The reference curve should be drawn along the boundary of outer segments of the photoreceptor layer. The reference curve is manually inserted, since its edges are frequently not clear.

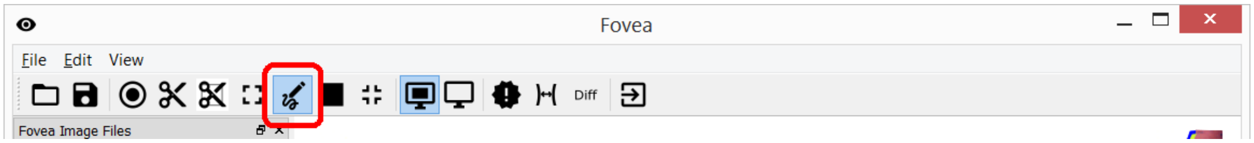

Click on the above icon to add the reference curve. Double-clicking mouse left button will add a point for the reference curve. The reference curve is the collection of lines that connects all points. You may need to do “double-click” several times to create a fitting reference line.

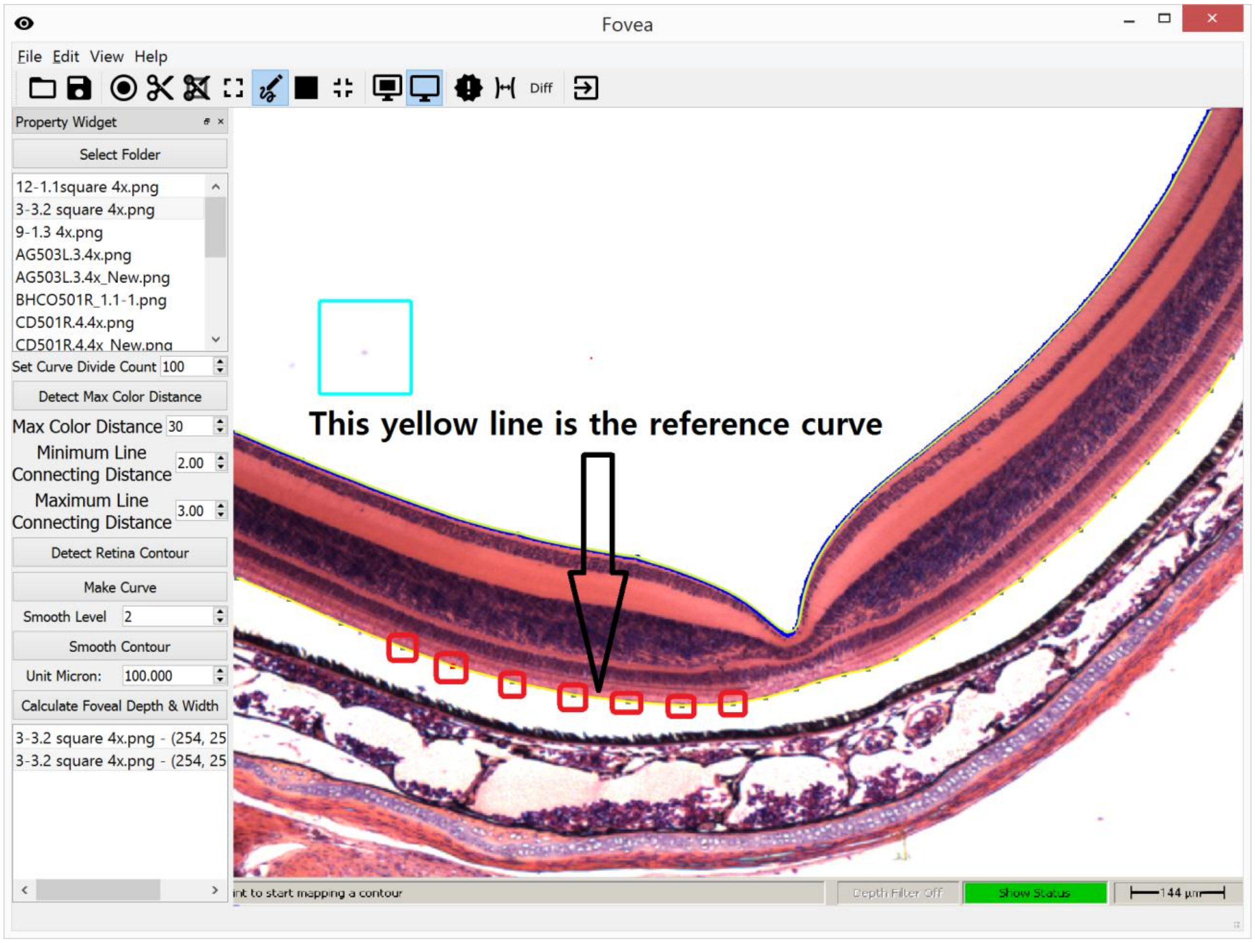

In the above image, the green dots and yellow curve encompass the reference curve. Users can add as many points as they wanted to refine the reference line.

6. Calculating the foveal width and depth

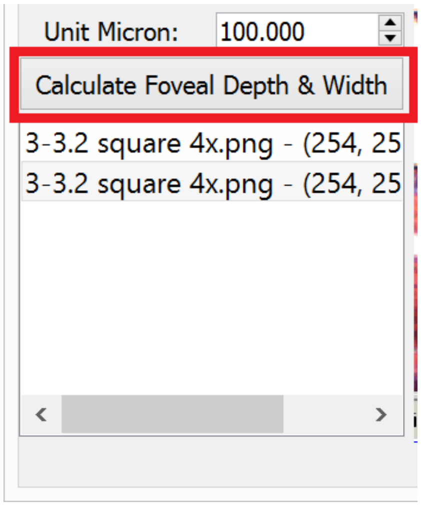

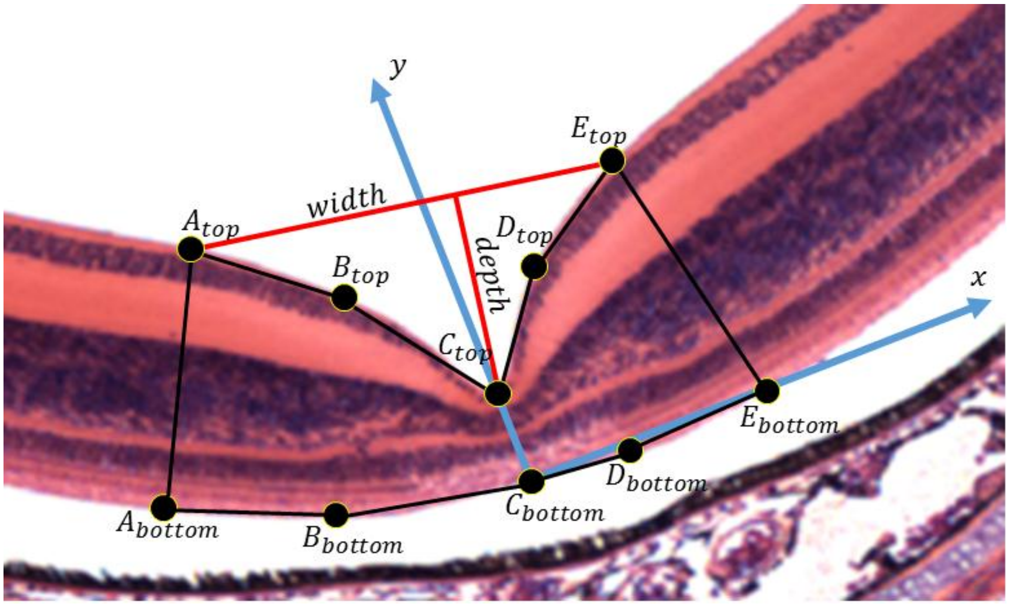

By pressing “detect depth and width” the application calculates the foveal depth and width.

It is done in the following way

1) The points A_{top}, C_{top}, and $E_{top}$are calculated as well as their counterparts on the bottom denoted A_{bottom}, C_{bottom}, and E_{bottom}.

2) The retina is then put in the center of the coordinate system and de-rotated. We put the point $C_{bottom}$to the center of the coordinate system and the y-axis is aligned to the line given by points $C_{bottom}$and C_{top}.The x-axis points to the right. Note, it does *not* need to go through the point E_bottom and in the image above it is just accidental.

3) Then the points B_{top} and B_{bottom}as well as $D_{top}$and D_{bottom} are calculated. They are significant points for necessary to determine the curvature of the fovea.

4) The *foveal width* is then calculated as the distance of points $A_{top}$and E_{top}.

5) The *foveal depth* is the distance of the point C_top from the line given by points A_{top} and E_{top}.

6) In the next step, the slope of the various points on is calculated. The slope is the angle given by a line and the x-axis of the coordinate system.

    a. *Local slope* is the angle between the tangent vector in a point and the x-axis. It is calculated for all *top* points.

    b. *Line slope* is the angle given by a pair of points and the x-axis and it is calculated for pairs $A_{top}B_{top}$, $B_{top}C_{top}$,$C_{top}D_{top}$, and $D_{top}E_{top}$.

7. Add Unit Measurement

The images are in the pixel space and they need to be converted to actual micrometers. To add the measurement unit, select in the icon below.

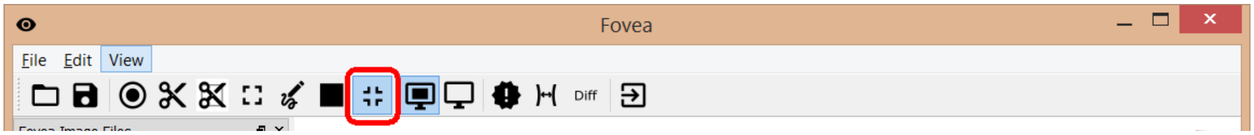

Most of fovea images show their own unit scale in the image. In the right corner of the below image, the unit measurement is presented (144 micrometer in this case). By simply drawing the line over the unit and typing the value, the pixels will be converted to the actual distance in micrometers

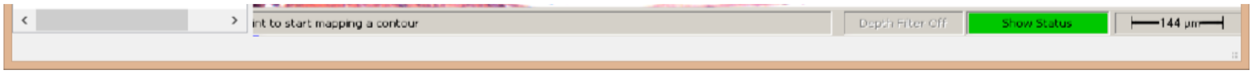

Users can add the unit measurement by "mouse left clicking and dragging".

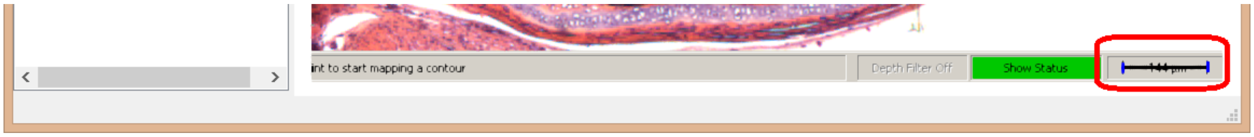

Then, in the property widget, we type that the actual micron distance of indicated line (red box in the image below).

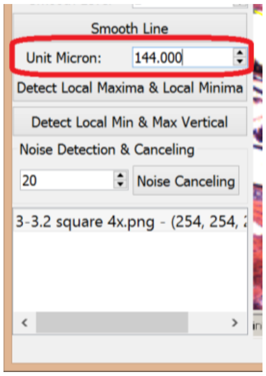

8. Saving the Result

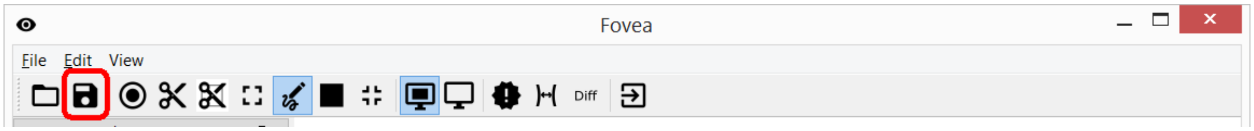

The result will be saved as a text file by clicking the icon above. The content looks like the following image.

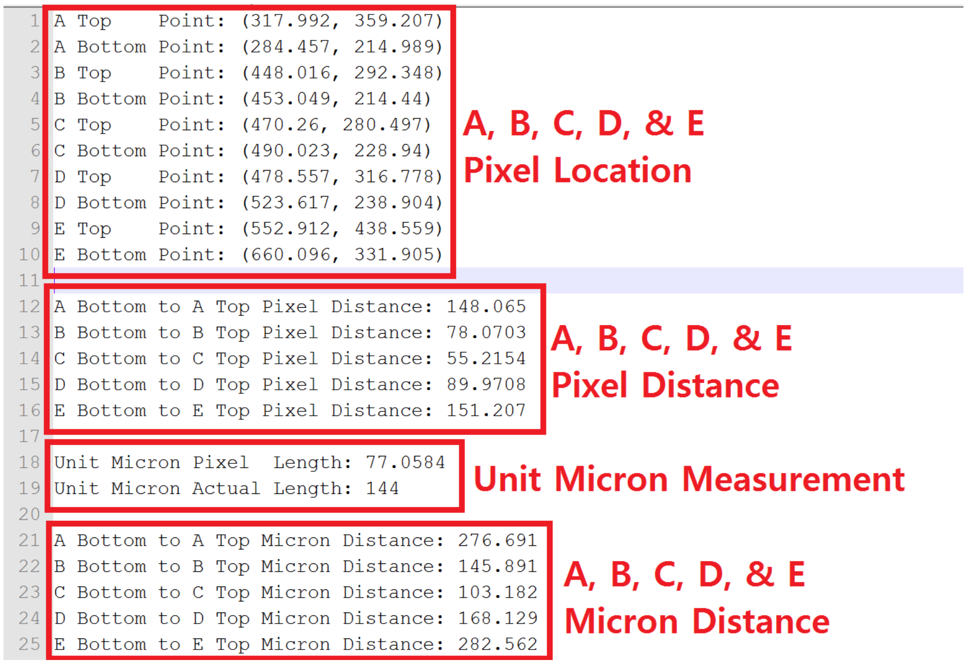

```
A Top    Point: (317.992, 359.207)
A Bottom Point: (284.457, 214.989)
B Top    Point: (448.016, 292.348)
B Bottom Point: (453.049, 214.44)
C Top    Point: (470.26, 280.497)
C Bottom Point: (490.023, 228.94)
D Top    Point: (478.557, 316.778)
D Bottom Point: (523.617, 238.904)
E Top    Point: (552.912, 438.559)
E Bottom Point: (660.096, 331.905)

A Bottom to A Top Pixel Distance: 148.065
B Bottom to B Top Pixel Distance: 78.0703
C Bottom to C Top Pixel Distance: 55.2154
D Bottom to D Top Pixel Distance: 89.9708
E Bottom to E Top Pixel Distance: 151.207

Unit Micron Pixel  Length: 77.0584
Unit Micron Actual Length: 144

A Bottom to A Top Micron Distance: 276.691
B Bottom to B Top Micron Distance: 145.891
C Bottom to C Top Micron Distance: 103.182
D Bottom to D Top Micron Distance: 168.129
E Bottom to E Top Micron Distance: 282.562
```


A, B, C, D, and E's pixel and micron distances are shown in the above image.

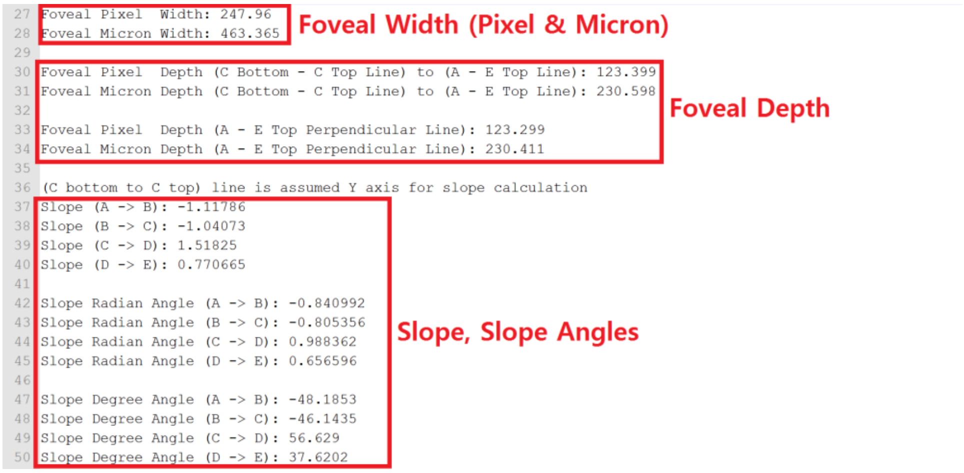

```
Foveal Pixel  Width: 247.96
Foveal Micron Width: 463.365

Foveal Pixel  Depth (C Bottom - C Top Line) to (A - E Top Line): 123.399
Foveal Micron Depth (C Bottom - C Top Line) to (A - E Top Line): 230.598

Foveal Pixel  Depth (A - E Top Perpendicular Line): 123.299
Foveal Micron Depth (A - E Top Perpendicular Line): 230.411

(C bottom to C top) line is assumed Y axis for slope calculation
Slope (A -> B): -1.11786
Slope (B -> C): -1.04073
Slope (C -> D): 1.51825
Slope (D -> E): 0.770665

Slope Radian Angle (A -> B): -0.840992
Slope Radian Angle (B -> C): -0.805356
Slope Radian Angle (C -> D): 0.988362
Slope Radian Angle (D -> E): 0.656596

Slope Degree Angle (A -> B): -48.1853
Slope Degree Angle (B -> C): -46.1435
Slope Degree Angle (C -> D): 56.629
Slope Degree Angle (D -> E): 37.6202
```


Also, the output stores information about the slope (tangent vector angle wrt to the x- axis) around each point as well as slope of points AB, BC, CD, and DE.

9. Additional Information

There is additional information that the program provides.

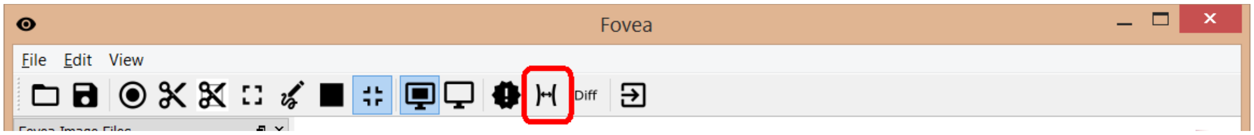

The icon above shows the "distance graph". The distance graph shows distance from the retina contour to the reference curve (pixel unit). The red dots indicate points A and E. Green dots show points B and D. Blue dot is the point C. If the distance graph is not continuous and is not smooth they user may need to redraw the reference curve or smooth the retina contour.

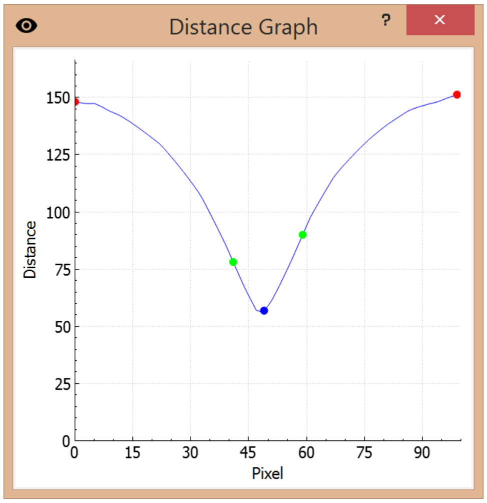

The "differentiation graph" (icon below) shows the second order derivative of the original graph.

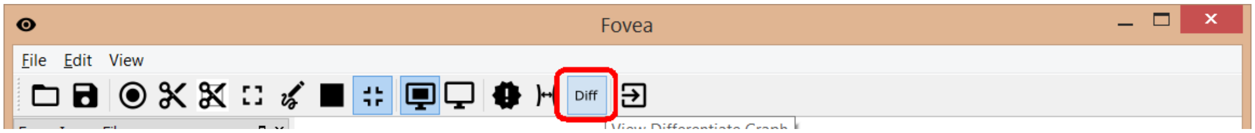

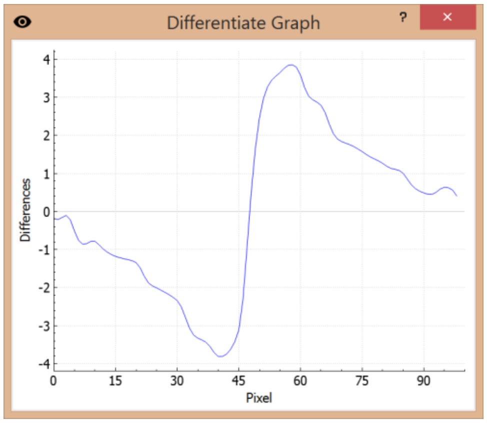

Supplement: Appendix S1 [file peerj-04-1785-s002.pdf]
